# Outbreak of febrile gastroenteritis caused by *Listeria monocytogenes* 1/2a in sliced cold beef ham, Italy, May 2016

**DOI:** 10.2807/1560-7917.ES.2018.23.10.17-00155

**Published:** 2018-03-08

**Authors:** Cristiana Maurella, Silvia Gallina, Giuseppe Ru, Daniela Adriano, Alberto Bellio, Daniela Manila Bianchi, Laura Chiavacci, Maria Ines Crescio, Margherita Croce, Valeria D'Errico, Maria Franca Dupont, Alessandro Marra, Ubaldo Natangelo, Francesco Pomilio, Angelo Romano, Stefano Stanzione, Teresa Zaccaria, Fabio Zuccon, Maria Caramelli, Lucia Decastelli

**Affiliations:** 1 Istituto Zooprofilattico Sperimentale del Piemonte, Liguria e Valle D'Aosta, Turin, Italy; 2 Igiene degli Alimenti e della Nutrizione (Food hygien and Nutrition Service) Local Health Unit TO4, Ciriè, Italy; 3 Istituto Zooprofilattico Sperimentale dell'Abruzzo e del Molise, National Reference Centre for Listeria monocytogenes, Teramo, Italy; 4 Laboratorio Microbiologia e Virologia U Città della Salute di Torino, Turin, Italy

**Keywords:** Listeria monocytogenes, outbreak investigation, food-borne infections, zoonoses

## Abstract

In May 2016, two separate clusters of febrile gastroenteritis caused by *Listeria monocytogenes* were detected by the local health authority in Piedmont, in northern Italy. We carried out epidemiological, microbiological and traceback investigations to identify the source. The people affected were students and staff members from two different schools in two different villages located in the Province of Turin; five of them were hospitalised. The epidemiological investigation identified a cooked beef ham served at the school canteens as the source of the food-borne outbreak. *L. monocytogenes* was isolated from the food, the stools of the hospitalised pupils and the environment of the factory producing the cooked beef ham. All isolates except one were serotype 1/2a, shared an indistinguishable PFGE pattern and were 100% identical by whole genome sequencing (WGS). By combining a classical epidemiological approach with both molecular subtyping and WGS techniques, we were able to identify and confirm a *Listeria* gastroenteritis outbreak associated with consumption of sliced cold beef ham.

## Introduction

*Listeria monocytogenes* is a ubiquitous Gram-positive food-borne pathogen that causes listeriosis both in humans and in several animal species. In some groups (people with weakened immune systems, older adults, newborns, pregnant women and their unborn babies), the disease can be an important cause of life-threatening septicaemia and meningoencephalitis [1]. Food-borne transmission of *L. monocytogenes* can also cause a self-limited acute febrile gastroenteritis, primarily reported among healthy people.

*L. monocytogenes* is widely distributed in natural environments and has been isolated from soil, decaying vegetation, stream water, sewage, urban environments and human and animal faeces [2]. Its ubiquity and ability to adapt and to survive under environmentally stressed conditions make it challenging to control and eradicate *L. monocytogenes* in food-processing environments and constitute a concern for the food industry [2]. The role of *L. monocytogenes* in food-borne outbreaks has been clearly recognised in the last decades [3-6]. Since 1993, listeriosis has been a reportable disease in Italy.

## Outbreak description

On 5 May 2016, five children from School A, an institution that includes two scholastic levels, a nursery with pupils aged 3–6 years and a primary school with pupils aged 6–10, and located in village A, a municipality of the province of Turin, were taken to the paediatric emergency room for symptoms of acute febrile gastroenteritis. On the same day, 26 of 162 (16%) pupils from the nursery and 25 of 400 (6%) pupils from the primary school returned home early (some of them before lunch time) because of fever, vomiting and abdominal pain. A report of a suspected food-borne outbreak was sent by the paediatric emergency room to the local health authority. In the following 3 days, additional pupils and staff from School A became ill including one hospitalised. On 6 May, the inspectors from the local health authority went to the mass catering food service (Caterer A) that provides meals to School A and collected retention samples (i.e. representative samples of the full meal stored by the mass catering food service) from the meals served on 3, 4 and 5 May 2016.

From 25 to 29 May 2016, 20 days after this first cluster was identified, children from School B, located in village B in the same Province, reported gastrointestinal symptoms similar to those in the first cluster. Once again, the local health authority inspectors collected retention samples from the mass catering food service (Caterer B, different from the previous one) that provided meals to School B.

We present the results of epidemiological, microbiological and traceback investigations for source attribution.

## Methods

### Case finding and hypothesis generation

An investigation team was created involving personnel from the local health authority, the hospital microbiology laboratory of Turin (Città della Salute) and the regional branch of the Istituto Zooprofilattico Sperimentale (IZS) in Turin as coordinating unit, dealing with both epidemiology and food analysis.

A standardised trawling questionnaire for gastrointestinal illness including questions on clinical symptoms, canteen attendance and foods eaten, was distributed by the local health authority to all the pupils’ parents and to staff of Schools A and school B. Administration of the questionnaire was postponed 20 days for the cluster associated with School B because of school holidays. At the IZS, questionnaire data were entered in an ad hoc database and analysed using Stata 14.1 [7] software.

Foods and environmental samples were submitted to the IZS laboratories for pathogen detection and quantification, and faecal samples were sent to the microbiology laboratory of the local hospital.

### Case definition

We initially defined a probable case as an attendee (pupils or staff) of School A or School B presenting with at least two of the following symptoms: headache, nausea, vomiting, diarrhoea, abdominal pain, temperature above 38 °C and an onset date starting from the day before the peak of the associated cluster. This definition served as the basis to build the epidemic curve for both clusters of the outbreak.

Individuals who reported symptom onset 2 days before the peak of the associated cluster were excluded from the case definition to favour specificity over sensitivity, and because information on foods eaten at this time was not available.

After a preliminary cohort analysis in School A, and to further increase specificity, we amended the case definition for the first cluster and defined a probable case as an attendee of School A presenting with at least three of the above-mentioned symptoms. This narrower operational case definition was not applied to the second cluster as there could have been a recall bias on the number of symptoms because of the time lag between the illness and the interviews.

We also used a final case definition for a confirmed case: an attendee of School A or School B presenting clinical symptoms and whose diagnostic test on stool samples confirmed the presence of *L. monocytogenes*.

### Cohort study and statistical analysis

We conducted a retrospective cohort study and calculated the attack rate (AR) and the relative risk (RR) for each food item. Confidence interval was set at 95% (95% CI).

For the first cluster, statistical analysis was performed both on the overall School A dataset and at the nursery and primary school level, used as a proxy for age. Only exposure data regarding 3 and 4 May were taken into account as the epidemic peak occurred on 5 May 2016 and it seemed unlikely that the meal served on that date, or later, could be the source of infection.

For the second cluster, the same exclusion criteria were applied and the data analysis referred to the meals consumed on 25 and 26 May.

### Microbiological investigation and traceback

#### Retention samples, other food samples and environmental samples 

Retention samples were sent to the food control laboratory of the IZS for quantification of *Bacillus cereus,* beta-glucuronidase-positive *Escherichia coli*, *Clostridium perfringens*, *Enterobacteriaceae*, coagulase-positive staphylococci, *Listeria monocytogenes and* sulfite-reducing anaerobic bacteria by routine methods. Samples were also tested for *B. cereus* diarrhoeal and emetic toxins, norovirus, *Salmonella* spp*.,* Shiga toxin-producing *E. coli*, and staphylococcal enterotoxins.

Regarding the first cluster and following preliminary analysis of the data, a specific food item was suspected as the source of the outbreak. Hence the local health authority was requested to trace-back the retailer and the producer to collect additional samples of the suspected food. Therefore, two weeks after the peak, one more food sample, an unopened package from a different batch, was collected from the producer (a cured meat factory) who had supplied the suspected food to the mass catering food service.

Regarding the second cluster, only retention samples from the meal served on 26 May 2016 were available at the mass catering food service, and an unopened package of the suspected food item from the same batch as the one served was retrieved from the producer. The same producer supplied the two mass catering food services involved in the two clusters. Environmental samples were collected pre-moistened sampling cellulose sponge bags (Solar-Cult, Solar Biologicals Inc, Vancouver, Canada) from surfaces that were not in contact with food at the mass catering food service that served School B and at the producer who had supplied food to both mass catering services.

#### Stool samples

Stool specimens were collected from the five hospitalised children (four from the first cluster and one from second cluster) and cultured using specific culture media for pathogenic bacteria (*B. cereus, Campylobacter* spp., *L. monocytogenes, Salmonella* spp., Shiga toxin-producing *E. coli, Shigella* spp., *Staphylococcus aureus,* and *Yersinia enterocolitica*). Characteristic colonies were only on *Listeria* medium (PalcamAgar, Liofilchem, Italy). *Listeria* strains were identified by MALDI-TOF technology.

### Identification of the *Listeria monocytogenes* strains

All *L. monocytogenes* strains isolated from stool, food and environmental samples were identified according to the ISO 11290 method using the Vitek MS system (bioMérieux, Marcy l'Etoile, France) and serotyped based on agglutination reactions with antisera for *L. monocytogenes* (Denka Seiken Co., Tokyo , Japan).

Pulsed-field gel electrophoresis (PFGE) was performed by the National Reference Laboratory for *L. monocytogenes* in Teramo, according to the United States Centres for Disease Control and Prevention PulseNet protocol [8], and analysed with Applied Maths BioNumerics software package (Version 7.5, Applied Maths, Saint-Martins-Latem, Belgium).

### Whole genome sequencing

Whole Genome Sequencing (WGS) of the DNA extracted from *L. monocytogenes* isolates was performed on the MiSeq platform (Illumina, San Diego, United States), using paired-end libraries which were preparated by following the Nextera XT DNA Library Preparation Kit (Illumina, San Diego, United States), with 200-bp read length. The reads were first subjected to FastQC Read Quality reports (Galaxy Version 0.65, accessed via the Galaxy public server at https://usegalaxy.org) [9] to provide the quality control checks on raw sequence data. We used Assembler 1.2 (accessed via Technical University of Denmark, DTU Center; https://cge.cbs.dtu.dk/services/Assembler/) to assemble genomes [10]. All samples were processed for multilocus sequence typing (MLST) with MLST 1.8 (accessed via https://cge.cbs.dtu.dk/services/MLST) [10]. A phylogenic comparison was made with CSI Phylogeny 1.2 (accessed via https://cge.cbs.dtu.dk/services/CSIPhylogeny) [11]. The single-nucleotide polymorphism (SNP)-typing phylogenetic method was used because of its high discriminatory power that allows distinguishing very closely related isolates to a degree not achievable by other typing methods [12]. SNP was performed with the following parameters: 10 × minimum depth at SNP position, 10% minimum relative depth at SNP position, 100 bp minimum distance between SNPs, 20 for minimum SNP quality, 20 for minimum read mapping quality, 1.98 minimum Z-score for each SNP, and including *L. monocytogenes* (GenBank accession number: AL591824.1) as reference.

## Results

### Epidemiological investigations

**Figure 1:**
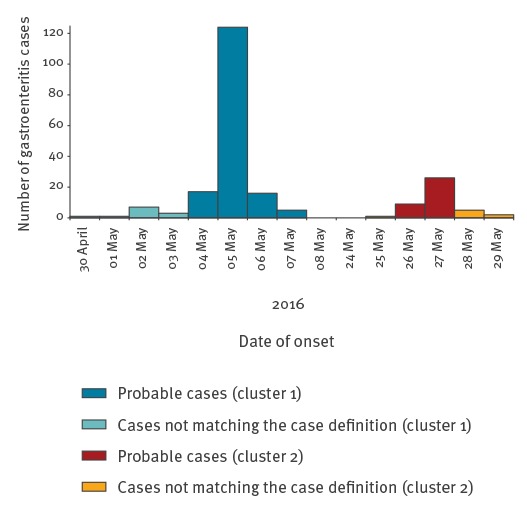
Epidemic curve of gastrointestinal illness in two schools, *Listeria monocytogenes* outbreak, Italy, May 2016 (n = 217)

The epidemic curve of the whole outbreak is shown in Figure 1. For each cluster, the pattern was compatible with a point-source epidemic.

The plot shows a classical point-source epidemic. Bars indicate probable cases by date of onset.

In the investigation of the first cluster, 484 completed questionnaires (response rate: 91.7%) were collected and used to build the cohort study. Symptoms were reported by 174 persons; 162 of 484 (33.7%) matched the probable case definition, while 11 people were excluded from the analysis. Reported symptoms among the 162 were: abdominal pain (n = 122; 75%), fever (n = 110; 68%), headache (n = 102; 63%), nausea (n = 85; 52%), diarrhoea (n = 79; 49%) and vomiting (n = 47; 29%). Two probable cases were staff members. All five probable cases who went to the hospital were afterwards confirmed.

Eight retention samples were analysed. As the microbiological analysis did not give any positive results, the output of the first cluster cohort analysis were used to hypothesise the most likely source of infection. ARs and RRs (Table 1) showed a positive association between consumption of a ‘beef ham’ (sliced cold beef meat, cured-cooked and dressed with oil and lemon) and the disease (RR = 2.23; 95% CI: 1.30–3.84). The association between the beef ham and the disease was still significant when stratifying the analysis by school level (nursery vs primary school) (Table 1). Moreover, the strength of the association further increased by using the narrower operational case definition (RR = 3.5; 95% CI: 1.5–8.3). 

**Table 1:** Attack rates among exposed and unexposed groups, and relative risks by food item consumed, by school level and day, *Listeria monocytogenes* outbreak, Italy, May 2016 (n =484)

Food	Exposed			Unexposed			RR	95% CI
	Total	Cases	% AR	Total	Cases	% AR		
**Two days before peaking **								
School A								
Pasta with tomato sauce	357	128	35.85	76	22	28.95	1.24	0.85-1.81
Halibut	338	126	37.28	95	24	25.26	1.48	1.02-2.14
Courgettes	202	74	36.63	231	76	32.90	1.11	0.86-1.44
Bread	328	112	34.15	105	38	36.19	0.94	0.70-1.27
Fresh fruit	277	105	37.91	156	45	28.85	1.31	0.98-1.75
Water	409	143	34.96	24	7	29.17	1.20	0.63-2.27
Nursery (pupils aged 3-6)								
Pasta with tomato sauce	293	93	31.74	62	15	24.19	1.31	0.82-2.10
Halibut	270	87	32.22	85	21	24.71	1.30	0.87-1.96
Courgettes	165	55	33.33	190	53	27.89	1.19	0.92-1.67
Bread	259	77	29.73	96	31	32.29	0.92	0.65-1.30
Fresh fruit	216	74	34.26	139	34	24.46	1.40	0.99-1.98
Water	336	104	30.95	19	4	21.05	1.47	0.61-3.56
Primary school (pupils aged 6-10)								
Pasta with tomato sauce	64	35	54.69	14	7	50.0	1.09	0.62-1.93
Halibut	68	39	57.35	10	3	30.0	1.91	0.73-5.04
Courgettes	37	19	51.35	41	23	56.10	0.92	0.60-1.39
Bread	69	35	50.72	9	7	77.78	0.65	0.43-0.99
Fresh fruit	61	31	50.82	17	11	64.71	0.79	0.51-1.2
Water	73	39	53.42	5	3	60.00	0.89	0.42-1.88
** One day before peaking**								
School A								
Vegetables soup	272	119	43.75	72	24	33.33	1.31	0.92-1.87
**Beef ham**	**290**	**132**	**45.52**	**54**	**11**	**20.37**	**2.23**	**1.30-3.84**
Roasted potatoes	286	114	39.86	58	29	50.00	0.8	0.59-1.07
Bread	267	107	40.07	77	36	46.75	0.86	0.65-1.13
Fresh fruit	242	103	42.56	102	40	39.22	1.09	0.82-1.44
Water	333	139	41.74	11	4	36.36	1.15	0.52-2.53
Nursery (pupils aged 3-6)								
Vegetables soup	205	85	41.46	59	17	28.81	1.44	0.93-2.22
**Beef ham**	**224**	**93**	**41.52**	**40**	**9**	**22.50**	**1.85**	**1.02-3.35**
Roasted potatoes	224	84	37.50	40	18	45.00	0.83	0.57-1.22
Bread	194	70	36.08	70	32	45.71	0.79	0.58-1.08
Fresh fruit	176	69	39.20	88	33	37.50	1.05	0.75-1.45
Water	257	99	38.52	7	3	42.86	0.9	0.38-2.14
Primary school (pupils aged 6-10)								
Vegetables soup	67	34	50.75	13	7	53.85	0.94	0.54-1.64
**Beef ham**	**66**	**39**	**59.09**	**14**	**2**	**14.29**	**4.14**	**1.13-15.16**
Roasted potatoes	62	30	48.39	18	11	61.11	0.79	0.51-1.24
Bread	73	37	50.68	7	4	57.14	0.89	0.45-1.75
Fresh fruit	66	34	51.52	14	7	50.00	1.03	0.58-1.83
Water	76	40	52.63	4	1	25.00	2.11	0.38-11.65

AR: attack rate; CI: confidence interval; RR: relative risk.

Significant associations are shown in bold.

A significant RR > 1 was found between the disease and the consumption of halibut (RR = 1.48; 95% CI: 1.02–2.14) for the initial case definition. However, this association disappeared after stratification by school (Table 1). When using the narrower case definition, halibut consumption again did not have a statistically significant association with the cases (RR = 1.18; 95% CI: 0.76–1.84), hence we considered it a chance finding and did not proceed to investigate it further.

In the investigation of the second cluster, 382 questionnaires were collected (response rate: 86.8%), although the questionnaires were administered with a delay of 20 days because of school holidays. Of these, 30 were excluded because data were not complete, and 352 were used for the cohort study analysis. Of those 352, 43 (12%) matched the probable case definition, with a peak of the outbreak on 27 May 2016 (Figure 1). The frequency of symptoms among those 43 was similar to the first cluster: abdominal pain (n = 34) headache (n = 25) fever (n = 23), nausea (n = 22), diarrhoea (n = 17) and vomiting (n = 13). We did not find any statistically significant associations, although the beef ham was the food item with the highest RR (RR = 1.4; 95% CI: 0.62–3.15; total responses: 268; 34 cases among 215 exposed and six cases among 53 unexposed).

### Microbiological investigations

Based on the results of the epidemiological analysis of the first cluster, a microbiological analysis was carried on both the retention sample and on the additional unopened package of the beef ham from the same batch as the one served in school A (batch no. 127529). Moreover, a retention sample and an unopened package belonging to the same batch as the one served in School B (batch no. 130578) from the second cluster were analysed, along with stools specimens from both the clusters and environmental samples from the shared producer (Table 2).

**Table 2:** Microbiological results of stool, food and environmental samples, *Listeria monocytogenes* outbreak, Italy, May 2016 (n = 23)

Cluster	Number of tested samples	Material sampled	Sampling place	Qualitative analysis (in 25 g)	Quantitative analysis	Serotype	PFGE profile (*Asc*I/*Apa*I)	MLST
1	1	Beef ham/retention sample (batch no. 127529)	Mass catering food service school A	Absence	< 10 cfu/g	ND	ND	ND
1	1	Beef ham/unopened batch (no. 127529)	Producer	Presence	> 15,000 cfu/g	1/2a	GX6A16.0119 / GX6A12.0305	ST11
1	4	Stool specimens	Hospital	Presence	ND	1/2a	GX6A16.0119 / GX6A12.0305	ST11
2	1	Beef ham/retention sample (batch no. 130578)	Mass catering food service school B	Presence	15,000 cfu/g	1/2a	GX6A16.0119 / GX6A12.0305	ST11
2	1	Beef ham/unopened batch (no. 130578)	Producer	Absence	< 10 cfu/g	ND	ND	ND
2	5	Environmental samples	Mass catering food service school B	Absence	ND	ND	ND	ND
2	1	Stool specimen	Hospital	Presence	ND	1/2a	GX6A16.0119 / GX6A12.0305	ST11
1 and 2	7	Environmental samples	Producer	Absence	ND	ND	ND	ND
1 and 2	1	Environmental sample (cutter machine)	Producer	Presence	ND	1/2a	GX6A16.0119 / GX6A12.0305	ST11
1 and 2	1	Environmental sample (fridge handle)	Producer	Presence	ND	1/2b	GX6A16.0024 / GX6A12.0306	ST5

cfu: colony-forming units; MLST: multilocus sequence type; ND: not done; PFGE: pulsed-field gel electrophoresis.

As a result, nine of 23 samples tested positive for *L. monocytogenes*. These were five stool samples (four from the first cluster and one from the second cluster), two food samples (one unopened package from a different batch obtained during the investigation of the first cluster and one retention sample from the second cluster, both with a count exceeding 15,000 colony-forming units (cfu)/g) and two environmental samples from the incriminated producer’s premise (one from the cutter machine and one from the fridge handle). 

### Identification of the *Listeria* strain

**Figure 2:**
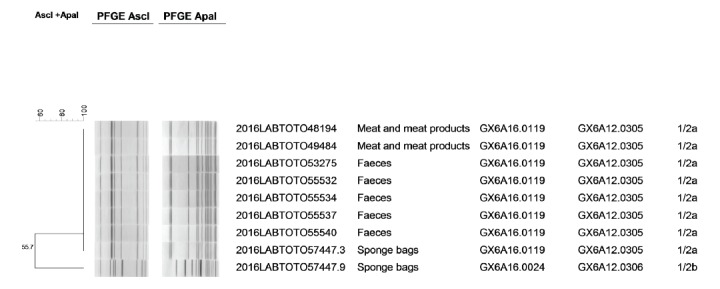
Dendrogram showing genetic similarity and genomic clusters of *Listeria monocytogenes* strains isolated from sliced beef ham, stools and environmental samples, Italy, May 2016 (n = 9)

Both the unopened batch (no. 127529) of beef ham collected during the investigation of the first cluster and the beef ham from the retention samples collected during the investigation of the second cluster tested positive for *L. monocytogenes*, with > 15,000 cfu/g and 15,000 cfu/g, respectively. In addition, seven *L. monocytogenes* isolates were obtained from the stool specimens from five children and from two environmental samples from the producer. Agglutination reactions assigned eight isolates to serotype 1/2a, while the remaining one (from the producer) was serotype 1/2b. In addition, all serotype 1/2a isolates showed indistinguishable PFGE pattern whereas the serotype 1/2b strain showed a different PFGE profile (Figure 2 and Table 2).

Clusters are indicated on the side of the figure. The identification numbers of isolates, origin of the samples, *Asc*I and *Apa*I profiles and serotypes are listed in the columns.

### Whole genome sequencing data analysis

**Figure 3:**
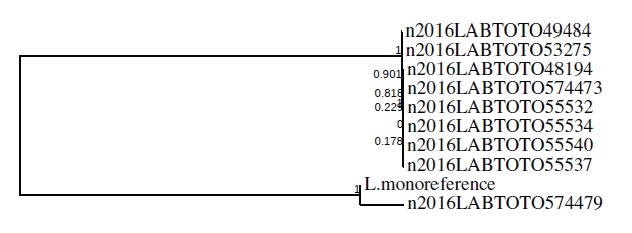
Single-nucleotide polymorphism analysis of *Listeria monocytogenes* isolates, Italy, May 2016 (n = 9)

In silico MLST showed that all serotype 1/2a isolates belonged to ST11 and the 1/2b isolate was ST5 (Table 2). The maximum likelihood tree obtained through the SNPs analysis showed two distinct clusters. The first cluster included eight ST11 isolates and the second cluster included the reference genome and the environmental ST5 isolate (Figure 3).

The maximum likelihood tree done with the CSI Phylogeny 1.2 tool highlights two distinct clusters, one that includes eight ST11 isolates and one that includes the ST5 environmental isolate and the reference strain “L. monoreference” (GenBank accession number: AL591824.1).

## Discussion

By combining an epidemiological approach and molecular typing including WGS techniques, we were able to identify and confirm a *Listeria* outbreak associated with the consumption of one food item, sliced cold beef ham.

The outbreak consisted of two school-associated clusters of non-invasive listeriosis cases linked to one *L. monocytogenes* strain. Cases experienced mostly abdominal pain, fever, headache and nausea. Gastrointestinal symptoms occurred rapidly after consumption of the incriminated meals, probably because a large dose of *L. monocytogenes* was ingested. A short incubation period for *L. monocytogenes*-associated gastroenteritis varying from 6 to 240 hours has already been reported elsewhere [13].

This outbreak was characterised by a high level of contamination of the suspected food. Unfortunately, it was not possible to determinate the exact number of *L. monocytogenes* in the beef ham sampled during the investigation of the first cluster because the laboratory in charge of official control had not expected a very high level of contamination and therefore only performed the dilutions needed in compliance with the Commission Regulation (EC) no. 2073/2005 to determine the threshold of *L. monocytogenes* < 100 cfu/g. It was not possible to repeat the analysis because it is mandatory to discard the samples after microbiological investigation. Isolation of a matching strain from the cutter machine suggests that the food contamination probably occurred in the production plant.

Another remarkable feature of the outbreak was the quite long persistence of the contamination. Norton et al. showed that specific *L. monocytogenes* ribotypes persisted over time in the environments of two of three processing plants for smoked fish [14]. Similar findings have been reported by a variety of groups that showed persistence of specific *L. monocytogenes* subtypes in different processing plants for smoked fish, poultry, meat or dairy [15-17]. In our investigation, the time span was 20 days from the first isolation of *Listeria* in the beef ham (unfortunately the date of production of the contaminated batch was not available) and the isolation from the environment of the cured meat factory where the beef ham was produced. This further confirms that *L. monocytogenes* can persist in the environment.

Among the 13 known serotypes of *L. monocytogenes*, the ones most frequently associated with human listeriosis are 1/2a, 1/2b and 4b, representing more than 95% of the infections. Our results are consistent with previous reports suggesting that serotype 1/2a is predominant in *L. monocytogenes-*associated gastroenteritis outbreaks [18], whereas serotype 4b was the most frequent up to 2010 and 4b is still the most reported serotype in invasive listeriosis outbreaks [19,20].

Although *L. monocytogenes* was not found in the retention sample of the first cluster, the epidemiological investigation was helpful in identifying a strong association between the consumption of beef ham and the disease. This association triggered the targeting and retrieval of a specific food for further microbiological analysis. A possible but weak association with another food, halibut, was seen in a first analysis based on the initial case definition, but not in stratified analysis. When using the narrowed case definition, it was considered a chance finding and no traceback was performed for halibut. Findings from the cohort study of the second cluster, although not statistically significant, were consistent and supported the same hypothesis. As shown by the inconclusive results from the epidemiological investigation of the second cluster, a stand-alone epidemiological approach may suffer from problems associated with the data collection or from a small sample size when the affected individuals come from a small community. In the second cluster, distribution and collection of questionnaires had to be carried out with some delay (20 days after the peak of the outbreak) leading to incomplete answers and probably recall problems. Consequently, there was the potential for non-differential misclassification of the exposure. That, combined with the small sample size, may explain our failure to obtain a statistically significant association. 

However, microbiological, molecular typing and WGS data helped confirm the link between the clusters that appeared to be independent based on the spatial distance (two different municipalities), the 20-day interval between them and the different catering suppliers. Historically, food-borne disease outbreaks of local scale, often linked to a single restaurant or social event, are caused by pathogens other than *Listeria* (e.g. *Salmonella*), even if *Listeria* have also been reported in connection with local outbreaks [21]. This is one example of a short *Listeria* outbreak that can also be responsible for scattered epidemics. Today, outbreaks often involve food products that are centrally produced and widely distributed geographically. In our case, two different mass catering food suppliers that served a number of schools scattered over many municipalities were supplied by a unique cured meat factory.

The problems in identifying the epidemiological links were successfully addressed through an integrated approach including questionnaire-based studies, trace-back investigations and biomolecular techniques. In the current study, the use of WGS made it possible to link human cases that occurred over a period of 20 days, brought about the understanding that there was in fact a single continuous outbreak source, and established links between two different mass catering food suppliers and one producer.

The correlation between food, stool and environmental specimens found in WGS, MLST and the phylogenetic analysis based on SNPs provided sufficient evidence for the local authorities to act accordingly and recall the contaminated food to prevent more outbreak-related cases. The authorities were also advised to monitor the follow-up of people exposed to the incriminated beef ham as it has been reported that severe listeriosis can have a long incubation period.

The output of the analysis from the first cluster highlighted a heavier impact in 6–10 year-old pupils than in younger ones, associated with a modification of effect between the exposure to *L. monocytogenes* and the age of the children. This may be explained by larger servings offered to the older pupils who probably experienced the highest exposure.

## Conclusion

The current study stresses the importance of an integrated approach when dealing with public health issues and suggests the usefulness of diagnostic techniques that enable clustering of isolates from different episodes.

## References

[1] Jordan K, Leong D, Ordònez Alvarez A. Listeria monocytogenes in the food processing Environment. SpringerBriefs in food, health and nutrition. Springer; 2015. p. 3-4.[REMOVED IF<> FIELD][REMOVED COMPARE<> FIELD][REMOVED IF<> FIELD]

[2] Ryser ET, Marth EH, editors. Listeria, listeriosis and food safety. 3rd ed. New York: CRC Press; 2007.[REMOVED IF<> FIELD][REMOVED COMPARE<> FIELD][REMOVED IF<> FIELD][REMOVED IF<> FIELD]

[3] Scallan E Hoekstra RM Angulo FJ Tauxe RV Widdowson MA Roy SL Foodborne illness acquired in the United States--major pathogens. Emerg Infect Dis. 2011;17(1):7-15. [REMOVED IF<> FIELD][REMOVED COMPARE<> FIELD][REMOVED IF<> FIELD][REMOVED IF<> FIELD] 10.3201/eid1701.P11101 21192848 PMC3375761

[4] Goulet V Hebert M Hedberg C Laurent E Vaillant V De Valk H Incidence of listeriosis and related mortality among groups at risk of acquiring listeriosis. Clin Infect Dis. 2012;54(5):652-60. [REMOVED IF<> FIELD][REMOVED COMPARE<> FIELD][REMOVED IF<> FIELD][REMOVED IF<> FIELD] 10.1093/cid/cir902 22157172

[5] Sauders BD D’Amico DJ Listeria monocytogenes cross-contamination of cheese: risk throughout the food supply chain. Epidemiol Infect. 2016;144(13):2693-7. [REMOVED IF<> FIELD][REMOVED COMPARE<> FIELD][REMOVED IF<> FIELD][REMOVED IF<> FIELD] 10.1017/S0950268816001503 27435307 PMC9150401

[6] Goulet V Lepoutre A Rocourt J Courtieu A-L Dehaumont P Veit P Epidemie de listeriose en France. Bilan final et resultats de l’enquete epidemiologique. [Listeriosis epidemic in France. Final assessment and results of the epidemiological survey]. Bull Epidemiol Hebdomadaire. 1993;4:13-4. French.[REMOVED IF<> FIELD][REMOVED COMPARE<> FIELD][REMOVED IF<> FIELD][REMOVED IF<> FIELD]

[7] StataCorp. Stata/SE version 14.1. College Station: StataCorp LP; 2016. Available from: www.stata.com[REMOVED IF<> FIELD][REMOVED COMPARE<> FIELD][REMOVED IF<> FIELD][REMOVED IF<> FIELD]

[8] Centers for Disease Control and Prevention (CDC). Standard operating procedure for PulseNet PFGE of Listeria monocytogenes. Atlanta: CDC; 2013. Available from: https://www.cdc.gov/pulsenet/pdf/listeria-pfge-protocol-508c.pdf[REMOVED IF<> FIELD][REMOVED COMPARE<> FIELD][REMOVED IF<> FIELD][REMOVED IF<> FIELD]

[9] Afgan E Baker D van den Beek M Blankenberg D Bouvier D Čech M The Galaxy platform for accessible, reproducible and collaborative biomedical analyses: 2016 update. Nucleic Acids Res. 2016;44(W1):W3-10. [REMOVED IF<> FIELD][REMOVED COMPARE<> FIELD][REMOVED IF<> FIELD][REMOVED IF<> FIELD][REMOVED IF<> FIELD] 10.1093/nar/gkw343 27137889 PMC4987906

[10] Larsen MV Cosentino S Rasmussen S Friis C Hasman H Marvig RL Multilocus sequence typing of total-genome-sequenced bacteria. J Clin Microbiol. 2012;50(4):1355-61. [REMOVED IF<> FIELD][REMOVED COMPARE<> FIELD][REMOVED IF<> FIELD][REMOVED IF<> FIELD] 10.1128/JCM.06094-11 22238442 PMC3318499

[11] Kaas RS Leekitcharoenphon P Aarestrup FM Lund O Solving the problem of comparing whole bacterial genomes across different sequencing platforms. PLoS One. 2014;9(8):e104984. [REMOVED IF<> FIELD][REMOVED COMPARE<> FIELD][REMOVED IF<> FIELD][REMOVED IF<> FIELD] 10.1371/journal.pone.0104984 25110940 PMC4128722

[12] Leekitcharoenphon P Kaas RS Thomsen MCF Friis C Rasmussen S Aarestrup FM snpTree--a web-server to identify and construct SNP trees from whole genome sequence data. BMC Genomics. 2012;13(Suppl 7):S6. [REMOVED IF<> FIELD][REMOVED COMPARE<> FIELD][REMOVED IF<> FIELD][REMOVED IF<> FIELD] 10.1186/1471-2164-13-S7-S6 23281601 PMC3521233

[13] Goulet V King LA Vaillant V de Valk H What is the incubation period for listeriosis? BMC Infect Dis. 2013;13(1):11. [REMOVED IF<> FIELD][REMOVED COMPARE<> FIELD][REMOVED IF<> FIELD][REMOVED IF<> FIELD] 10.1186/1471-2334-13-11 23305174 PMC3562139

[14] Norton DM McCamey MA Gall KL Scarlett JM Boor KJ Wiedmann M Molecular studies on the ecology of Listeria monocytogenes in the smoked fish processing industry. Appl Environ Microbiol. 2001;67(1):198-205. [REMOVED IF<> FIELD][REMOVED COMPARE<> FIELD][REMOVED IF<> FIELD][REMOVED IF<> FIELD] 10.1128/AEM.67.1.198-205.2001 11133446 PMC92546

[15] Nesbakken T Kapperud G Caugant DA Pathways of Listeria monocytogenes contamination in the meat processing industry. Int J Food Microbiol. 1996;31(1-3):161-71. [REMOVED IF<> FIELD][REMOVED COMPARE<> FIELD][REMOVED IF<> FIELD][REMOVED IF<> FIELD] 10.1016/0168-1605(96)00978-6 8880305

[16] Rørvik LM Caugant DA Yndestad M Contamination pattern of Listeria monocytogenes and other Listeria spp. in a salmon slaughterhouse and smoked salmon processing plant. Int J Food Microbiol. 1995;25(1):19-27. [REMOVED IF<> FIELD][REMOVED COMPARE<> FIELD][REMOVED IF<> FIELD][REMOVED IF<> FIELD] 10.1016/0168-1605(94)00080-P 7599028

[17] Senczek D Stephan R Untermann F Pulsed-field gel electrophoresis (PFGE) typing of Listeria strains isolated from a meat processing plant over a 2-year period. Int J Food Microbiol. 2000;62(1-2):155-9. [REMOVED IF<> FIELD][REMOVED COMPARE<> FIELD][REMOVED IF<> FIELD][REMOVED IF<> FIELD] 10.1016/S0168-1605(00)00395-0 11139016

[18] Althaus D Lehner A Brisse S Maury M Tasara T Stephan R Characterization of Listeria monocytogenes strains isolated during 2011-2013 from human infections in Switzerland. Foodborne Pathog Dis. 2014;11(10):753-8. [REMOVED IF<> FIELD][REMOVED COMPARE<> FIELD][REMOVED IF<> FIELD][REMOVED IF<> FIELD] 10.1089/fpd.2014.1747 25007293

[19] Stephan R Althaus D Kiefer S Lehner A Hatz C Schmutz C Foodborne transmission of Listeria monocytogenes via ready-to-eat salad: A nationwide outbreak in Switzerland, 2013-2014. Food Control. 2015;57:14-7. [REMOVED IF<> FIELD][REMOVED COMPARE<> FIELD][REMOVED IF<> FIELD][REMOVED IF<> FIELD] 10.1016/j.foodcont.2015.03.034

[20] Swaminathan B Gerner-Smidt P The epidemiology of human listeriosis. Microbes Infect. 2007;9(10):1236-43. [REMOVED IF<> FIELD][REMOVED COMPARE<> FIELD][REMOVED IF<> FIELD][REMOVED IF<> FIELD] 10.1016/j.micinf.2007.05.011 17720602

[21] Pichler J Much P Kasper S Fretz R Auer B Kathan J An outbreak of febrile gastroenteritis associated with jellied pork contaminated with Listeria monocytogenes. Wien Klin Wochenschr. 2009;121(3-4):149-56. [REMOVED IF<> FIELD][REMOVED COMPARE<> FIELD][REMOVED IF<> FIELD][REMOVED IF<> FIELD] 10.1007/s00508-009-1137-3 19280142

