# Addendum for Euro Surveill. 2018;23(10)

**DOI:** 10.2807/1560-7917.ES.2018.23.44.181101

**Published:** 2018-11-01

**Authors:** Eurosurveillance editorial team

**Affiliations:** 1 European Centre for Disease Prevention and Control (ECDC), Stockholm, Sweden

In the article entitled ‘Outbreak of febrile gastroenteritis caused by Listeria monocytogenes 1/2a in sliced cold beef ham, Italy, May 2016‘ by C Maurella et al., published on 8 March 2018, GenBank accession numbers were added on 30 October 2018 at the request of the authors:

The Listeria monocytogenes​ whole genome sequences assembled have been deposited at DDBJ/ENA/GenBank under the BioProject accession no. PRJNA436467 and the single accession numbers related to each sample are: QUNX00000000, QUOE00000000, QUNZ00000000, QUOD00000000, QUOC00000000, QUOA00000000, QUOB00000000, QUOB00000000, RCYX00000000; the version described in this paper is version .1 for all genomes.

**Table:** *Listeria monocytogenes​* whole genome accession numbers, Italy, May 2016

Whole genome accession number​	BioSample ID	Strain ID
QUOE00000000	SAMN09230455	2016LABTOTO55534
QUNZ00000000	SAMN09230460	​2016LABTOTO48194
QUOD00000000	SAMN09230456	​2016LABTOTO55537
QUOC00000000	SAMN09230457	​2016LABTOTO53275
QUOA00000000	SAMN09230459	​2016LABTOTO55532
QUNY00000000	SAMN09230461	​2016LABTOTO574473
QUOB00000000	SAMN09230458	​2016LABTOTO55540
QUNX00000000	SAMN09230462	​2016LABTOTO574479
RCYX00000000	SAMN09230454	​2016LABTOTO49484

